# Editorial Comment: Flexible ureterorenoscopy and laser lithotripsy with regional anesthesia vs general anesthesia: A prospective randomized study

**DOI:** 10.1590/S1677-5538.IBJU.2019.0770.1

**Published:** 2020-09-02

**Authors:** John Denstedt

**Affiliations:** 1 Division of Urology Western University Ontario Canada Division of Urology, Western University, Ontario, Canada

## COMMENT

The authors report on results of a randomized prospective trial in comparable groups of patients with intrarenal stones treated by flexible ureteroscopy and laser lithotripsy ( 1 ). Patients were randomized to either general or regional epidural anesthesia with the primary outcome measures being surgeon comfort and other standard measureable results of fURS treatment. After exclusions or intraoperative conversion to general anesthesia or PCNL, 45 patients in the RA group were compared to 61 in the GA group.

They find no significant difference in OR times, hospital stay, postoperative pain, fluoroscopy times or stone free rates defined as 2mm or smaller fragments on non contrast CT one month post treatment.

The sentinel finding in this study was an increased complication rate in the regional anesthesia group comprising patients with either bradycardia, mucosal tear or hemorrhage. They attribute the mucosal injuries and bleeding to a lack of good control of laser energy secondary to respiratory excursion of the kidney in patients under regional anesthesia. Moreover “surgeon comfort” which was subjectively measured during the procedure was also significantly less in procedures performed under regional anesthesia.

While the numbers are small these results are interesting and emphasize the point that intrarenal surgery with a flexible ureteroscope and laser is an operation of millimeters. Both as regards the stone fragmentation and in avoiding tissue injury. Stones in the mid to distal ureter are relatively unaffected by significant respiratory excursion while those in the kidney can be subject to substantive movement especially in the awake patient. It would have been desirable to have more information on the detailed questions in the surgeon comfort questionnaire and whether this is a validated tool. Nonetheless this study does inform us of some of the pros and cons of differing anesthesia methods for retrograde intrarenal surgery and thus discussions with patients and our anesthesia colleagues.
